# Accuracy of indocyanine green videoangiography in predicting visual outcomes for anterior skull base surgeries, compared to intraoperative visual evoked potential: a pilot explorative study

**DOI:** 10.3389/fneur.2026.1762604

**Published:** 2026-04-01

**Authors:** Fatemeh Khafaji, Bernardo Reyes Medina, Christoph Sippl, Stefan Linsler

**Affiliations:** Department of Neurosurgery, Medical Campus Upper Franconia, Klinikum Bayreuth Friedrich-Alexander University Erlangen-Nuremberg (FAU), Bayreuth, Germany

**Keywords:** ICG-videoangiography, intraoperative visual evoked potential, perichiasmatic tumor, perioptic mass lesions, skull base tumor

## Abstract

**Introduction:**

Preserving the microcirculation of the optic nerve (ON) during surgical decompression is crucial, yet there is no dependable intraoperative predictor of visual outcomes. This study examines the use of indocyanine green videoangiography (ICG-VA) as a potential predictor, comparing its effectiveness with intraoperative visual evoked potential (VEP).

**Methods:**

We analyzed 13 patients with various perichiasmatic pathologies. Each underwent enhanced MRI, CT scans, detailed ophthalmological examinations, and VEP. Five control patients were included, presumed to have normal pial support post-aneurysmal clipping. Indocyanine green (ICG) was administered at 0.2 mg/kg via peripheral venous access after a rapid injection of 10 mL of 0.9% NaCl before and after tumor resection, using flow analysis software (Flow 800, Carl Zeiss Co.). We meticulously measured the intervals for ICG’s appearance in the internal carotid artery (ICA) and the pial circulation of the optic nerve as well as the full saturation of both before and after resection.

**Results:**

The study included 13 patients and 5 controls (mean age: cases 55.30 ± 15.2 years; controls 57.8 ± 2.38, *p* < 0.05). Twelve patients had preoperative visual field impairment, with 10 showing postsurgical improvement. Prolonged P100 latency in VEPs was noted in 11 cases and 3 controls (case: 9.81 ± 13.11 ms; control: 5.9 ± 8.33 ms). Mean improvement in peak time of the optic nerve relative to the ipsilateral ICA was 0.82 ± 2.32 s post-resection (controls: 0.45 ± 0.87 s, *p* < 0.05). VEP yielded 60% sensitivity, 50% specificity, and 85.7% positive predictive value. ICG-VA demonstrated lower sensitivity (44%) but 100% specificity and positive predictive value. ROC analysis showed greater discrimination for ICG-VA (AUC = 0.790) than for VEP measures. No ICG-related complications occurred.

**Conclusion:**

ICG-VA of the optic nerve is a promising modality for predicting visual outcomes compared with VEP. Moreover, it could provide more details on the ON vasculature, which would help preserve it during tumor resection. However, additional research, including randomized trials and data, is required to establish a significant difference.

## 1 Introduction

Perioptic and perichiasmatic lesions of the skull base are prevalent pathologies in skull base surgery, where preserving vision is the primary goal of decompression procedures. Currently, visual evoked potential (VEP) is the only intraoperative monitoring method utilized. Key VEP components, specifically the P100 and N75 waveforms, indicate optic nerve (ON) function, enabling near-real-time monitoring during surgery (1). While VEP monitoring may enhance surgical outcomes, it is subject to debates, including anesthetic effects, intraoperative variables, and patient-specific differences. Moreover, VEP monitoring lacks real-time assessment capabilities, and its clinical utility remains to be thoroughly examined (2).

An emerging concept in this field emphasizes the need to understand the vascular dynamics of the ON. This understanding is essential for clinicians and researchers, particularly regarding various perioptic pathologies and interventions affecting ON integrity (3).

Intraoperative indocyanine green videoangiography (ICG-VA) of the ON has been reported in only two case studies (4, 5) and a pilot study (6).

Recent reports by Ceccato et al. (4) and Han et al. (5) highlight ICG-VA’s vital role in understanding the optic nerve’s vascular anatomy, especially in perioptic tumor resections and vascular compressive lesions. ICG-VA provides insights into ON perfusion, blood flow dynamics, and vascular abnormalities, essential for preserving vision during surgery. Ceccato et al. (4) show that ICG-VA aids in intraoperative vascular assessment and preoperative diagnosis, helping refine surgical decisions by outlining perfusion and risks. Han et al. (5) support these findings, demonstrating ICG’s effectiveness in visualizing the optic nerve’s complex vascular network tissues (4, 5). In 2024, the Osorio et al. (6) group validated and refined their method, injecting 5 mL ICG after endonasal endoscopic tumor removal. The absence of fluorescence within 10 s was found to be statistically significantly associated with postoperative visual impairment. None of these studies had reported VEP during the surgeries.

This original research represents the first comprehensive, standardized study assessing the preservation and beneficial effects of ICG-VA, as a adjunctive tool, in combination with VEP, on visual preservation during perioptic and perichiasmatic surgeries. Additionally, this study aims to investigate the clinical implications of ICG-VA in evaluating and managing pathologies associated with the ON. By integrating insights from Ceccato et al. (4) and Han et al. (5), this research intends to provide a comprehensive assessment of the advantages and limitations of ICG-VA across various clinical scenarios, with a particular emphasis on its potential to enhance surgical outcomes, improve diagnostic accuracy, and facilitate timely interventions for conditions such as tumor-induced compression.

## 2 Methods

This study is a prospective, non-randomized cohort investigation involving patients admitted for asleep surgery on intra-axial and extra-axial lesions in the perioptic and perichiasmatic areas. Five control subjects were selected from patients admitted for elective clipping of an aneurysm in the anterior circulation of the Willis circle. The study was conducted at the Neurosurgical Department of Bayreuth Hospital, Medical Campus of the Friedrich-Alexander-University Erlangen-Nuremberg in Upper Franconia. The Ethics Committee of the Friedrich-Alexander-University Erlangen-Nuremberg on Human Research approved this prospective study (No. 24-129-B). It was conducted in accordance with the ethical guidelines outlined in the Declaration of Helsinki. Patients provided signed consent for their participation.

Thirteen patients diagnosed with various perichiasmatic pathologies were selected from our department for this analysis. Each patient underwent a comprehensive imaging study, including magnetic resonance imaging (MRI) with and without contrast enhancement, a computed tomography (CT) scan of the skull base, and an ophthalmologic examination employing the Goldman perimetric standard and visual acuity. The ophthalmological exam was performed similarly before and after surgery.

Since the ophthalmological consultation was conducted by a different department, the examinations were performed thoroughly in the preoperative phase. The postoperative assessment typically focused on the preoperative deficits and their subsequent improvement. Due to heterogeneity in the deficits and a limited sample size, the primary visual outcome was categorized as either improved, unchanged, or deteriorated.

All patients underwent a supraorbital craniotomy for tumor resection and decompression of the optic nerve. One leading surgeon operated on all patients and controls (SL). The inclusion criteria encompassed individuals over 18 years of age, patients who provided signed consent, those with no known allergy to ICG, and those with no documented severe renal or thyroid insufficiency. The exclusion criteria were preoperative blindness, as assessed by an ophthalmologist; absence of a signed consent form; and individuals under 18 years of age. All control patients provided signed consent for their participation.

### 2.1 Visual evoked potential

The only anesthetic used was Total Intravenous Anesthesia (TIVA), including propofol (4 to 8 mg/kg/min), Remifentanil (1–2 μg/kg/min), and Atracurium (0.5 mg/kg). LED goggles were placed over the eyelids and secured with a non-transparent patch. Inomed stimulation devices operated at a pulse frequency of 1.1 Hz to reduce line hum in the VEP. The red LEDs emitted light at 654 nm, achieving up to 26,000 Lux, regulated to 10,000 Lux during surgery to ensure VEP viability. Subcutaneous corkscrew electrodes were inserted according to the international EEG 10–20 system on the occipital regions (O1, O2) and along the cortical midline (Cz, Fz). The VEP was recorded using the Inomed ISIS System with a four-channel montage at O1 and O2 relative to Fz and Cz. The VEP was band-pass filtered from 5 to 100 Hz over a 100 ms window. The high-frequency filter restricted components from 100 to 300 Hz. The N75 and P100 peaks were delineated within 50 to 150 ms post-stimulation. N75 amplitude was calculated as the voltage difference between N75 and P100 peaks, while P100 amplitude was the difference between P100 and the subsequent negativity. VEP monitoring began after patient setup, documenting at least two responses for reproducibility. Any substantial changes in latency or amplitude—10% latency delay or 50% amplitude reduction—would trigger an alert. VEP was recorded three times throughout the surgery.

The first VEP baseline data were documented before the skin incision. The second P100/N75 latency was recorded with the initial ICG-VA before tumor resection, and any amplitude reduction was noted. VEP was continuously monitored throughout surgery to identify changes, with final latencies (P100, N75) and amplitudes documented with the second ICG-VA after tumor resection. SSEPs of the median and tibial nerves were performed to ensure procedural feasibility.

### 2.2 ICG-videoangiography

The intraoperative administration of indocyanine green (ICG) was conducted intravenously at a dosage of 0.2 mg/kg, preceded by a rapid injection of 10 mL of 0.9% NaCl before and after tumor resection. Flow 800 software (Zeiss Meditec, Oberkochen, Germany) was used for ICG-VA analysis, offering semi-quantitative contrast enhancement assessments. This software analyses flow dynamics in ICG fluorescent angiography, displaying results as color-coded maps: high-flow arteries in red and low-flow vessels in blue. The ipsilateral internal carotid artery (ICA) served as a reference for the ON. The intervals between the initial ICG appearance in the ICA and its presence in the pial circulation of the ON were meticulously measured both pre- and post-resection. ICG-VA recordings for control patients were obtained immediately after aneurysm clipping, focusing on the ON in the surgical field. The region of interest (ROI) was delineated at the ICA and ON to ensure consistent measurements before and after tumor resection. Flow 800 generates a color-coded graphical representation detecting velocity and slope time within the ROIs. Multiple ROI measurements were collected using ImageJ Software (NIH), and intensity data for the ON and ICA were transferred to Excel for graphing. The ICG-VA recording was adjusted to eight frames per second for validation. The authors compared the maximum intensities of the optic nerve and ICA with those from Flow 800.

Statistical analyses were conducted using non-parametric tests with IBM SPSS Statistics 30 and GraphPad Prism Version 10.4.2, with statistical significance established at *p* < 0.05. The accuracy was calculated following the STARD Standard 2015 (7).

## 3 Results

The analysis included 13 tumor patients (7M, 6F) and 5 aneurysm patients (5F), with a mean age of 55.3 ± 15.20 yrs. and 57.8 ± 2.38 yrs., respectively (*p* < 0.05). The overall mean follow-up was 6.7 ± 3.25 months. The mean tumor volume was 7639.13 ± 9613.62 mm^3^. No patient suffered complications related to ICG injection post-surgically. Table 1 summarizes demographic features of both groups. The main pathologies included six anterior skull base meningiomas, one pituitary adenoma, three craniopharyngiomas, one pituitary metastasis, one glioma, and one pituicytoma. Visual outcomes improved/unchanged in 10 patients (*p* < 0.05). Visual deterioration occurred in a hypophyseal metastasis and a pituicytoma. The visual outcome remained unclear in one patient with meningioma due to ipsilateral CN-III paresis and respiratory insufficiency postoperatively. Depending on which anatomical region was infiltrated, we categorized the pathologies into four anatomical areas:

- Region I, the tumor invaded one side ON;
- Region II, the tumor infiltrated the chiasmatic area;
- Region III, the tumor infiltrated one ON and the chiasm; and
- Region IV tumor invaded both ONs and the supra/infrachiasmatic area.

**Table 1:** Demographic features of tumor patients and aneurysm patients, mean ipsilateral P100 shows delay from the baseline.

	Tumors	Controls	*p*-value
*N*	13 (7M, 6F)	5F	0.036
Age (year)	55.30 ± 15.2	57.8 ± 2.38	0.049
Tumor volume (xyz mm^3^)	7639.13 ± 9613.62		
Hb preoperative (gr/L)	14.03 ± 1.32	13.28 ± 1.8	0.443
Hb postoperative (gr/L)	12.43 ± 1.08	11.5 ± 1.35	0.577
Blood lost (mL)	295.45 ± 65.01	370 ± 248.99	0.023
OP duration (min)	207.84 ± 49.5	200 ± 84.16	
Peak time ICA-ON before tumor resection (sec)	2.48 ± 2.33	0.45 ± 0.87	0.027
Peak time ICA-ON after tumor resection (sec)	3.39 ± 2.47		
Mean time ICA-ON improvement (sec)	0.825 ± 2.32		
Mean ipsilateral P100 latency (ms)	9.81 ± 13.11	5.9 ± 8.33	0.862
Amplitude reduction >50%*	7/13	2/5	0.261

*Amplitude reduction of more than 50% during tumor resection or clipping.

**Figure 1:**
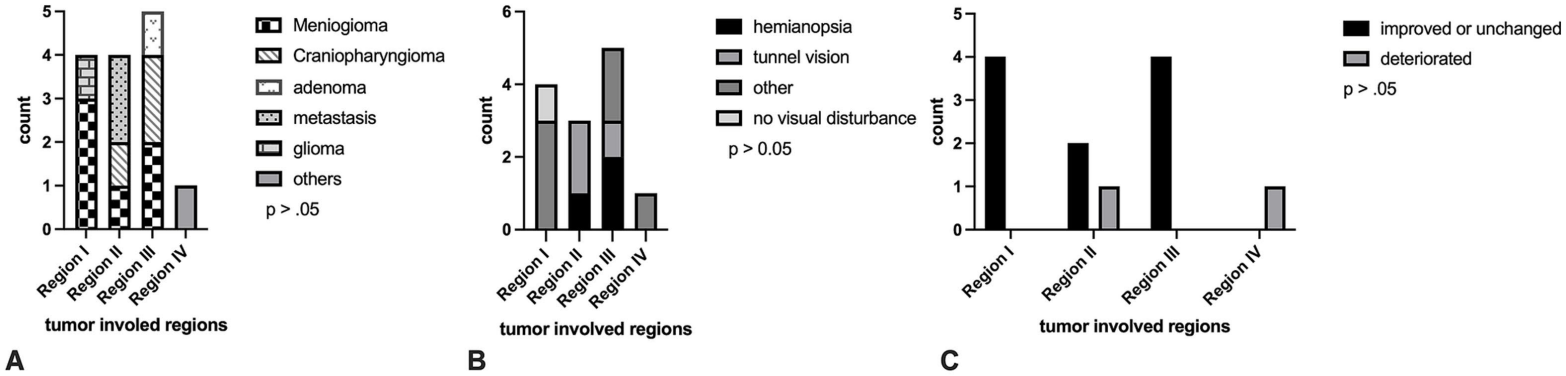
The distribution of tumor types **(A)**, their associated visual disturbances **(B)**, and ultimately their visual outcomes **(C)**, categorized by anatomical regions.

No significant correlation was found between the tumor-involved region, symptoms, and postoperative visual outcome. Region III exhibited a higher tumor mass (*p* > 0.05). Figure 1 illustrates the distribution of various pathologies, their primary ophthalmologic disturbances, and ultimately, their visual outcomes in comparison to the infiltrated regions.

### 3.1 ICG-videoangiography

**Figure 2:**
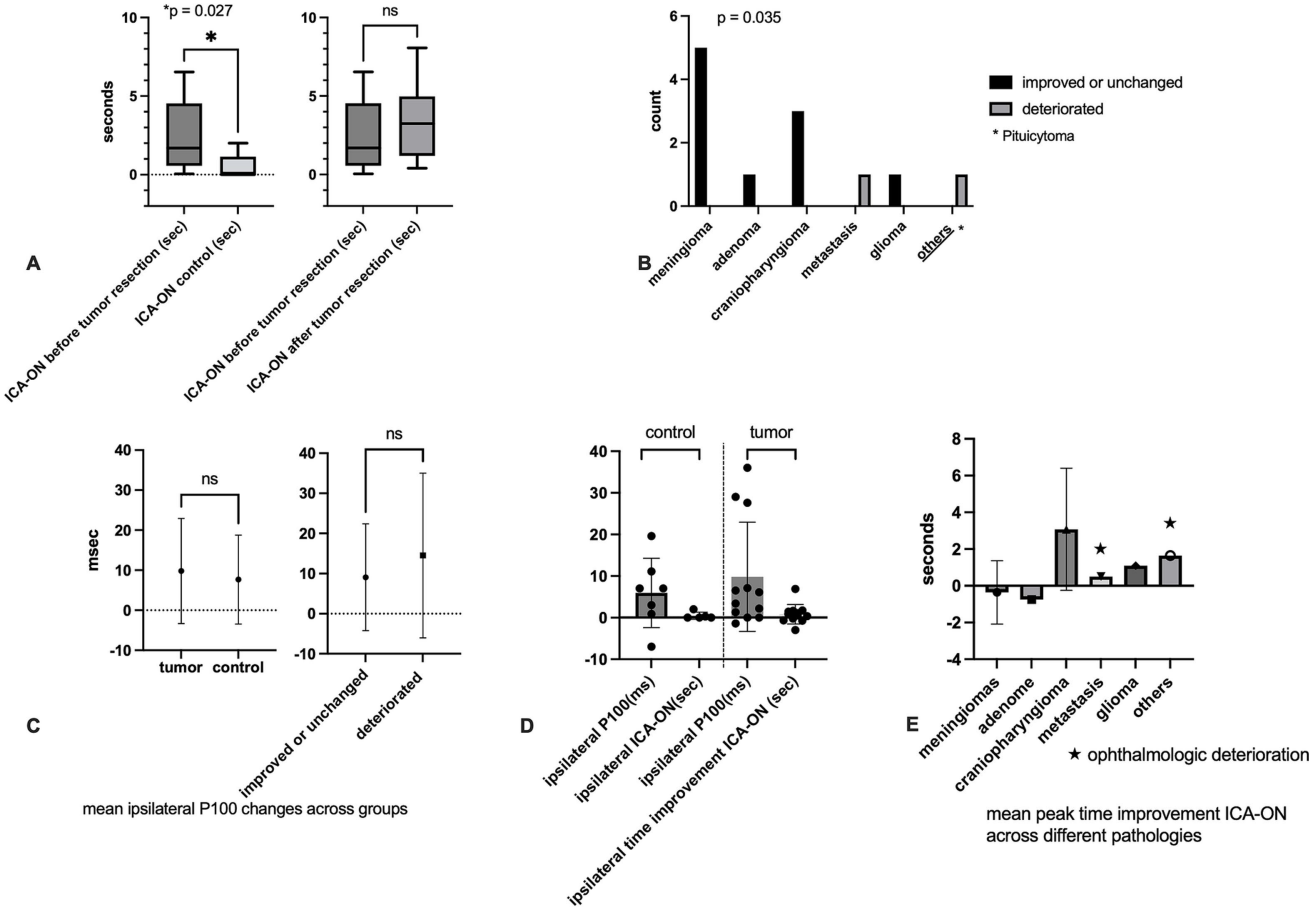
**(A)** Independent-sample Mann–Whitney *U* test ICA-ON peak time difference between tumors and controls at the base time (left), after tumor resection across tumor patients (middle), and ICA-ON peak time improvement between meningiomas and other pathologies (right). **(B)** Comparing different pathologies across ophthalmologic outcomes, meningiomas showed a significant better outcome. **(C)** Independent-sample Mann–Whitney *U* test P100 latency across tumors and controls (left) and ophthalmologic outcome (right); tumor patients and patients with unfavorable outcome demonstrated more prolonged latencies. **(D)** Independent-sample Mann–Whitney *U* test comparing the ipsilateral P100 latencies and ipsilateral ICA-ON peak time differences between tumors and controls. **(E)** Mean ± SD ICA-ON peak time improvement across different pathologies; meningiomas and adenoma depict better time improvement along with favorable outcome (ns, not significant).

The peak time differences of ICA-ON prior to tumor resection were recorded at 2.48 ± 2.33 s and subsequently at 3.39 ± 2.47 s following tumor resection (*p* > 0.05). The mean improvement in time from ON to ICA was 0.82 ± 2.32 s for all pathologies. The peak time difference for ICA-ON in the control group was 0.45 ± 0.87 s (*p* < 0.05). The peak time improvement is determined by subtracting the ICA-ON value post-tumor resection from the pre-resection ICA-ON measurement. Considering all pathologies together, a comparison of peak-time improvement in ICA-ON regarding outcomes showed no significant difference. A second analysis of all ICG-VA using ImageJ software confirmed the accuracy of the Flow 800 software measurements (Figure 2).

#### 3.1.1 ICG-VA of meningiomas

The meningioma, as the largest group infiltrating region I–III, exhibited a statistically significant time improvement of the ON-ICA following tumor resection, which correlated positively with the ophthalmological outcomes (Figures 2A,B). Figure 2E provides further details regarding the time improvement following tumor resection across various pathologies (see also Supplementary Video 1).

### 3.2 Visual evoked potential

The SSEP showed consistent stability and reliability during all surgeries, remaining uniform across tumor and aneurysm (control) groups. The P100 latency appeared prolonged during surgery in both groups and at the conclusion of tumor resection, in tandem with the second ICG-VA. For the ipsilateral eye, the mean P100 latencies were 9.81 ± 13.11 ms and 5.9 ± 8.33 ms for the tumor and aneurysm groups, respectively (*p* > 0.05).

As expected in a similar anesthesiologic condition for both groups, the P100 latencies were prolonged after the craniotomies. The most prolonged was observed after temporarily clipping the ipsilateral ICA for clipping Pcom aneurysm (32 ms). Nevertheless, no ICA-ON peak time difference was observed during the ICG-VA after clipping.

The mean prolongation of latency in P100 did not demonstrate any statistically significant difference between tumor and aneurysm, across ophthalmologic outcomes, or among the two primary tumor groups (meningiomas and craniopharyngiomas) characterized by favorable outcomes. Overall, VEP remained stable and reproducible during 13 surgeries (*p* > 0.05). The amplitude reduction, considered a predictive factor, was observed during surgery in 7 tumors and two aneurysms (*p* > 0.05). Only two tumor patients deteriorated postoperatively (Table 1; Figures 2C,D).

### 3.3 Calculation of accuracy

**Figure 3:**
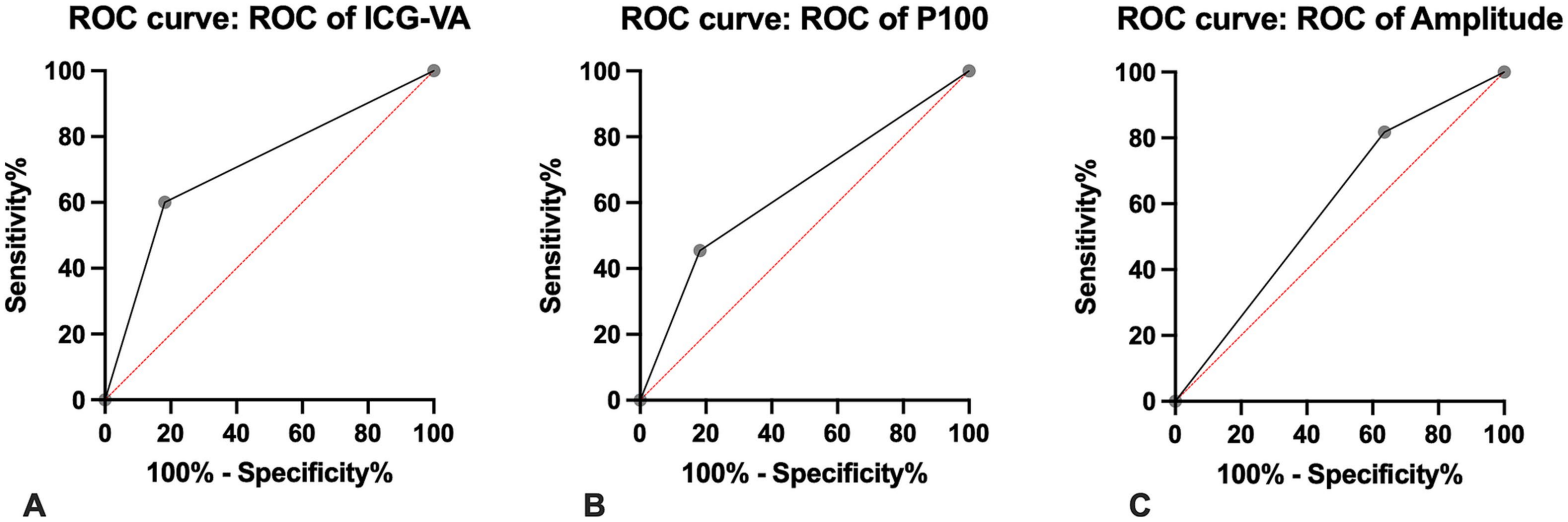
Receiver operating characteristic (ROC) curves for ICG videoangiography **(A)**, P100 latency **(B)**, and amplitude reduction **(C)**. The area under the curve (AUC) was highest for ICG (AUC = 0.790), followed by P100 latency (AUC = 0.636) and amplitude reduction (AUC = 0.590), indicating that ICG had the highest overall diagnostic performance.

True Positive (TP), True Negative (TN), False Negative (FN), and False Positive (FP) values were meticulously extracted. Calculating the confidence interval (CI) was unfeasible due to the insufficient number of patients. Table 2 compares the diagnostic performance of ICG-VA, P100 latency, and amplitude reduction. Amplitude reduction demonstrates maximal sensitivity (100%) but limited specificity (44%) and positive predictive value (PPV) (28.5%), indicating a high false-positive (NPV) rate. ICG videoangiography exhibits perfect specificity and PPV (100%) but low sensitivity (44%) and NPV (28.5%). P100 latency provides moderate sensitivity (60%), specificity (50%), and a high PPV (85.7%). ROC analysis reveals the greatest discriminative ability for ICG (AUC = 0.790), followed by P100 latency (AUC = 0.636) and amplitude reduction (AUC = 0.590). Figure 3 illustrates the ROC curves of the three modalities.

**Table 2:** Calculation of sensitivity, specificity, positive predictive value (PPV), and negative predictive value (NPV), and receiver operating characteristic (ROC).

	ICG video-angiography	Latency P100	Amplitude reduction
Sensitivity	44%	60%	100%
Specificity	100%	50%	44%
PPV	100%	85.7%	28.5%
NPV	28.5%	20%	100%
ROC	0.790	0.636	0.590

## 4 Discussion

This analysis represents the first comprehensive, standardized study comparing the preservation and beneficial effects of ICG-VA as a adjunctive tool alongside VEP on visual preservation during perichiasmatic neurosurgical procedures. Additionally, the authors described the clinical implications of ICG-VA in evaluating and managing pathologies associated with the ON.

The study presented almost identical populations between tumors and controls. Comparing demographic features, anesthesiologic factors, and surgical duration did not reveal significant differences between the patient and control groups, thereby validating comparable conditions for both cohorts. Nearly all procedures were performed by a single surgeon (SL), thereby enhancing the study’s accuracy and reliability. Meningiomas have shown substantial visual improvement following tumor resection and ON decompression. Our study indicated that any mass lesion compromises the microcirculation of the ipsilateral ON and chiasm, in contrast to the nearly normal condition observed in aneurysmal patients. The peak time difference between the ICA and ON was significantly longer prior to tumor resection than in the aneurysmal patients. After tumor resection, the peak time difference ICA-ON improved the most in meningiomas and adenomas; given the small sample size, it did not reach statistical significance. The enhancement of peak time was markedly more evident in instances of meningiomas (*p* > 0.05). However, it did not display any significant correlation with the ophthalmologic outcome. Conversely, craniopharyngiomas exhibited a paradoxical behavior. While the visual outcomes improved in all three cases following surgery, the peak time discrepancies between the ICA-ON persisted after tumor resection (Figures 2B,E). Intraoperative VEP, as a prognostic factor, yielded results comparable to those of other studies (2, 8). This discussion aims to explore the significance of VEP in surgeries involving the anterior optic tract, as well as the importance of ICG-VA in neurosurgical oncological procedures.

### 4.1 Visual evoked potential

Our study utilized VEP under near-normal conditions, such as aneurysm clipping. As in tumor groups, P100 prolongation was also detected in the aneurysm group, which did not show a statistically significant difference compared to the tumor group. It is essential to note that P100 latency tends to be prolonged shortly after dural opening. Amplitude reduction has been demonstrated to be a superior prognostic indicator during perichiasmatic surgeries. Nevertheless, there exists a paucity of studies that compare this factor in nearly normal cases. In our investigation, transient amplitude reduction was evident in the majority of instances during tumor resection or manipulation of the optic nerve.

VEP remains the only tool to monitor ON during skull base surgeries, but its accuracy is still questionable. The initial aspect to consider is that flash VEP primarily represents the macular function (9–11). Gutzwiller et al. (12) found intraoperative VEP has 85% sensitivity and 78% specificity in predicting postoperative visual outcomes. VEP changes better reflect visual acuity than visual field defects (1).

Feng et al. (13) found no significant correlation between latency and visual field outcomes; amplitude changes correlated more with the visual field improvement. Another study concluded that intraoperative VEP measurements do not correlate with visual field outcomes in patients with perioptic tumors (14). A retrospective study demonstrated that new major visual field defects might go undetected with VEP (15). The weak link between latency variations and visual field outcomes does not necessarily signify a technical failure, but rather an intrinsic physiological limitation, which accounts for the discrepancy between new postoperative visual field deterioration and perioperative derived VEP signal (16). Statistical analysis revealed no significant correlation between changes in intraoperative VEP waveforms and postoperative visual acuity or visual performance field (8, 14, 17, 18). Intraoperative VEP was also mentioned as an ineffective screening for visual acuity loss due to low sensitivity and high false positives (8). A further meta-analysis indicated that VEP exhibits a sensitivity of 47.2% for identifying visual deterioration and a sensitivity of 10% for detecting movement. The predictive value for improvement in vision stands at 25%, whereas the predictive value for deterioration is noted to be 60%. Additionally, the false predictive value for deteriorated VEP results is 40%, while the false predictive value for improved VEP results is 75% (2). Despite the utilization of TIVA, visual evoked potentials frequently exhibit unreliability and may yield misleading results (8, 17, 18). A case series indicated that VEP alterations during the surgical procedure exhibited a reproducibility rate of 89.6% (8). Other review articles reported the stability and reproducibility of VEP ranging from 83 to 100%; however, not all studies mentioned utilized standard LED goggles for intraoperative stimulation (19). Intraoperative VEPs are typically more predictive and reliable when tumor infiltration impacts the optic radiation in the parietal, temporal, or occipital lobes, or when employing direct cortical recording as opposed to conventional transcortical methods electrodes (20, 21). Numerous limitations are associated with intraoperative VEP, as documented in the existing literature. VEP demonstrates substantial variability among individuals and tends to exhibit relative instability or susceptibility, thereby constraining its wide-ranging clinical application (22). A key drawback of VEP is its age restriction; it is recommended for use by individuals aged 18 and not older than 60 (23). Like other monitoring methods, a limitation of the VEP is that preexisting visual field defects or anopsia can also diminish its effectiveness (24). Preoperative visual acuity or field deficits may disrupt intraoperative VEP elicitation (12, 25, 26). Finally, regarding the LED color, no notable difference was observed between the use of red LED and white LED (27). Some studies suggested no correlation between visual outcome and the alteration of VEP during surgery (14, 28). Conversely, certain studies reported a 100% positive predictive value for VEP. This group found that baseline intraoperative VEP values are strongly linked with preoperative visual function. As a result, a lower baseline amplitude suggests preoperative visual impairment. The significant intraoperative amplitude variability complicates accurate interpretation, occasionally leading to misinterpretation (29). Many studies agree that a more than 50% increase in amplitude predicts visual improvement, and vice versa (8, 29–32). Some studies emphasize that VEP recordings can only be obtained from patients without significant visual impairments (1, 8, 28, 33). When tumors like craniopharyngioma adhere to the optic nerve or chiasm, the presence of stable VEP often still leads to new visual deficits after surgery, affecting predictability (32, 34). Another aspect is the time needed to create a wave. Normally, VEP requires between 100 to 300 s to produce a wave. It is generally not predictive or practical for the anterior visual pathway, and a reduction in amplitude or loss of waves often occurs even during the extradural phase of the procedure (15).

Multiple studies identified tumor-related factors that independently predict visual outcomes alongside VEP parameters. Tight tumor adhesion to optic structures and a larger tumor volume emerged as consistent predictors across the craniopharyngioma studies (32, 35).

Studies varied markedly in their reporting of diagnostic accuracy metrics. Mattogno et al. (29) provided one of the most comprehensive quantitative assessments, demonstrating that perioperative VEP amplitude changes correlated significantly with postoperative visual acuity (*p* < 0.0001) and visual field changes (*p* = 0.0013). Tao et al. (36) defined optimal warning criteria through receiver operating characteristic analysis, identifying threshold values of 51.76% reduction for N75-P100 amplitude (AUC 0.816, *p* < 0.001) and 38.80% reduction for P100-N145 amplitude (AUC 0.738, *p* < 0.001). Both amplitude reduction ratios served as independent predictors for postoperative visual dysfunction in multivariate analysis (*p* < 0.001 and *p* = 0.018).

Our data indicate that the latency associated with P100 is often delayed even prior to the opening of the dura mater. As tumor resection commences, the amplitude exhibits a decrease but recovers spontaneously upon completion of the resection. However, a comparable pattern was noted in the control group. A comparison of the VEP data across various pathologies did not disclose any significant differences correlated to outcomes. As a result, we were unable to establish a substantial correlation with ophthalmological outcomes, including visual acuity and visual field.

Furthermore, variations in intensities, frequencies, or recording time settings were documented across the studies, leading to considerable variability in the results. Each study utilized a specific VEP setting, which evidently undermines the reliability of the VEP findings. Consequently, VEP necessitates further refinement and adjustments due to its sensitivity being below 50% (2).

### 4.2 ICG-videoangiography

No evidence exists comparing ICG-VA to VEP for predicting visual outcome during perioptic skull base surgeries, as all identified studies examined ICG exclusively without VEP (4–6, 37, 38). Indocyanine green (ICG) is a near-infrared fluorescent dye from the tricarbocyanine family, widely used in medical diagnostics. Following intravenous administration, ICG binds to plasma proteins, remaining in the intravascular system for 3 to 4 min before liver metabolism. It is significant in retinal diagnostics and microcirculation during neurovascular surgeries, potentially reducing the need for postoperative digital subtraction angiography (39–41).

Flow 800 software analyses flow dynamics in ICG fluorescent angiographic studies, offering semi-quantitative contrast enhancement analyses. Kamp et al. (42) describe Flow 800’s physics, creating 2D tissue maps of the surgical site that show maximal fluorescence or the half-maximal time. It measures fluorescence changes in ROIs, aiding detection of delayed perfusion in ischemic areas in various pathologies. Flow 800 provides objective graphical data, reducing interobserver variability. Several factors, including blood pressure, heart rate, ejection fraction, and injection rate, can also cause variations in results (43).

Conversely, administering ICG injections too closely together may lead to false-positive results, depending on how surgeons interpret them (44). A 2020 study used Flow 800 Software to predict hypoperfusion syndrome after bypass surgeries for Moya-Moya patients. Results showed delay time and slope metrics effectively predicted outcomes, despite only assessing ROI perfusion with Flow 800 (45).

Five studies examined the use of ICG angiography during skull base surgery to assess optic apparatus perfusion and predict visual outcomes (4–6, 37, 38). ICG methodology varied across studies, though common elements emerged. ICG dosing ranged from 5 mg to 12.5 mg, with the lower dose diluted in 10 mL saline and the higher dose administered as a bolus (6, 38). The studies demonstrated varying levels of evidence for ICG angiography’s predictive value. The most robust quantitative findings came from the 2024 Osorio et al. (6) study, which found that lack of chiasm fluorescence within 10 s of ACA fluorescence was significantly associated with new postoperative vision deficits (*p* = 0.005). The two case reports show improved pial circulation in the optic nerve after resection of a tuberculum sellae meningioma, as assessed by ICG-VA and associated with better visual outcomes. A 2016 report noted increased blood perfusion and ICG intensity post-resection. Ceccato et al. found that preserving ON vessels during surgery improved postoperative vision. *ImageJ* software was used to analyze both case studies (4, 5).

The presented investigation represents an explorative effort to employ a structured protocol to evaluate the effectiveness of ICG-VA, in conjunction with Flow 800 Software, in maintaining ON vascular integrity across varied pathologies. Results show differing effects of sellar lesions on ON pial circulation, and note that under near-normal conditions, there’s minimal peak difference between the ON and the ICA. This phenomenon may be due to the optic nerve’s primary vascular supply originating from the ICA’s first branches, specifically the superior hypophyseal and ophthalmic arteries (46).

Tumors affecting the perichiasmatic area can alter the vascular architecture of the ON, leading to delayed ICG filling compared with the ICA. Post-resection, meningiomas notably enhanced pial circulation of the ON, correlating with better ophthalmological outcomes. The primary mechanism underlying visual improvement is mechanical decompression of the optic nerve. Zevgaridis et al. (47) explicitly note that meningiomas in the supra- and parasellar region cause visual loss through optic nerve compression, and the presence of an intact arachnoid membrane around the lesion emerged as a highly significant, favorable prognostic factor (*p* < 0.001). Similar benefits were noted with adenomas. Nevertheless, other tumor types leading to poorer outcomes showed decreased pial circulation in the ON. Interestingly, craniopharyngiomas showed an opposing trend: despite favorable outcomes, the time difference between ICG peaks in the ICA and ON was prolonged. Bassiouni et al. (48) identify preservation of the microvasculature supplying the optic apparatus as a critical factor for visual improvement.

#### 4.2.1 Correlation between improvement of optic nerve perfusion and the axonal recovery

No study has demonstrated a direct correlation between a quantitative enhancement of optic nerve (ON) perfusion and both short-term and long-term axonal recovery. One study indicated that optic canal decompression results in over 90% improvement in vision, including visual acuity and visual field, thereby suggesting a potential connection between immediate perfusion restoration and subsequent axonal recovery. Interestingly, the late follow-up showed a visual outcome almost identical to that at postoperative follow-up (49). Another study indicated that surgical intervention may restore optic nerve functionality, potentially through the preservation of axons and the reversal of myelin loss. Nevertheless, it does not explicitly elucidate the mechanisms underlying immediate perfusion restoration or delayed recovery (50). It is evident from the literature that numerous questions remain to be addressed regarding the pathophysiology of optic nerve injury and the factors that affect recovery. An earlier study examined various optic neuropathies and suggested that optic nerve sheath decompression could reverse optic nerve ischemia, a prevalent pathophysiological feature across these conditions. This intervention may facilitate immediate restoration of perfusion and delayed axonal and functional recovery (51). Utilizing diffusion tensor imaging and optical coherence tomography to identify stages of ON injury following compression by any mass lesion includes conduction block, demyelination, ischemic insult, and retrograde and anterograde degeneration (52). Several animal studies have demonstrated the complexity of degeneration, regeneration, and delayed cell death signals at both intercellular and intracellular levels following optic nerve injury. In adult rats, experiments using specific markers of axonal transport and neurofilament restoration indicate that visual recovery occurs within about 3 weeks after optic nerve injury (53). Notably, axonal transport recovers at around 3 weeks, but this recovery coincides with a second wave of cell death rather than improved neuronal survival, as demonstrated in rats using *in vivo* confocal neuroimaging (54). Conversely, following axonal injury, intercellular signaling was initiated within 30 min, Catastrophic cell death signals within 6 hours from retinal ganglion cells (RGCs), followed by programmed cell death and a pro-apoptotic process (55).

Overall, an explorative study shows that there is no recovery period for axonal injury in the optic nerve of mammals, as vision loss becomes permanent once axons are damaged (56). Combining all together elucidates some of the intricacies involved in the mechanism and architecture of optic nerve injury.

#### 4.2.2 Correlation between the microcirculation of the optic nerve and neuronal

Considering the vascular supply of the optic nerve, the optic chiasm’s superior part is supplied by branches of the anterior cerebral and anterior communicating arteries, while its inferior part is mainly supplied by the internal carotid, basilar, posterior cerebral, and posterior communicating arteries. The central region receives blood only from this inferior network, and a separate lateral part is supplied by direct branches of the internal carotid artery (46). The inferior part of the chiasm has more blood vessels, which may explain quick recovery of bitemporal hemianopsia after pituitary decompression. During surgery, the inferior vessels are often distorted, indicating that visual field defects are mainly due to ischemia, not direct compression. The superior hypophyseal and posterior communicating arteries supply this area. Salaud et al. found no anastomosis in a cadaveric study (3, 43). The complexity of the pial blood supply of the optic apparatus and its associated pathologies has not been thoroughly addressed in the literature. However, the paradoxical behavior of the ICG-VA during craniopharyngioma surgeries may be elucidated by certain study findings. Research has documented a visual recovery rate of 47.1% following endonasal endoscopic surgery for craniopharyngioma. It should be noted that radical surgical procedures may jeopardize the vascular supply to the optic nerve and chiasm complex, potentially resulting in ischemic abnormalities and visual impairments (57). The vascular supply patterns of craniopharyngiomas, specifically the involvement of inferior chiasmatic branches, may contribute to bitemporal visual field defects attributable to hypoperfusion, indicating a potential association between vascular supply and the preservation of visual function during surgical resection (58). In summary, ICG-VA accurately depicts the real-time vascular dynamics of the optic nerve and chiasm both prior to and following tumor resection. The neuromolecular and neurofunctional aspects, along with their relationship to the vascular supply of the optic nerve remnants, remain areas of uninvestigated inquiry. It remains uncertain whether visual recovery or stabilization is contingent upon immediate perfusion enhancement. Additional research is essential to investigate the microvascular blood supply associated with various sellar and perioptic pathologies and their correlation to the pial blood supply of the optic pathway, particularly in relation to visual function, neuronal interactions, and flow dynamics. Such investigations are vital to advancing understanding and optimizing treatment strategies that preserve optic nerve integrity.

### 4.3 Limitations and outlook for the future

It seems that ICG-VA of the optic nerve could provide some benefits for the VEP. Our study employed a monocentric, prospective design with a limited patient cohort, which may introduce selection bias. The rarity of perichiasmatic lesions made it difficult to establish a randomized trial framework to assess ICG-VA’s efficacy in predicting visual outcomes.

Additionally, the small number of control patients limited the ability to identify distinct ICG-VA patterns among those with nearly normal vascularization. The visual outcomes of the control group were not assessed through comprehensive ophthalmological examinations, thereby restricting direct clinical evaluation and constraining the interpretation of specificity and negative predictive value correlations with prolonged P100 in patients with aneurysms. Subclinical or transient visual dysfunction cannot be ruled out. Moreover, only initial postoperative visual outcomes were documented, and long-term follow-up data are lacking. Nonetheless, it is unlikely that visual outcomes would worsen months post-surgery, as existing literature and clinical experience suggest that any deterioration in visual acuity and fields occurs immediately after the operation and is captured within the initial assessment. Our results, however, were comparable to those of similar studies (34, 59, 60). Our preliminary accuracy results are limited to interpretability due to the small patient sample size. Considering the low NPV, weak sensitivity, and the limited size of our cohort, we are unable to establish a stable predictive tool for the visual outcome following perioptic tumor resection. Intraoperative VEP, however, did not demonstrate superior efficacy. Combining both techniques could potentially improve visual outcomes, considering the limitations of each method, and warrants further refinement. During optic nerve decompression, combining techniques may provide better insights into the vascular supply of the optic nerve and tumor, possibly reducing delays and inconsistencies seen with VEP, which remains unstandardized. Changes proposed by research organizations include adjustments to LED settings in goggles and their frequencies. It is also important to note that approaches such as supraorbital or pterional may displace goggle LEDs, affecting VEP results but not ICG-VA. Clinical visual outcomes vary significantly, especially regarding intraoperative VEP reliability, with literature reporting predictive accuracy ranging from 100% to below 50%. Different research groups interpret VEP differently, some relying on amplitude, others on latency. VEP requires TIVA conditions and takes 100–300 s to produce waveforms, whereas ICG-VA provides circulation information immediately. Using Flow 800 software, it takes about 70 s to visualize velocity, slope, and differences between the ICA and ON. However, ICG-VA can only be performed a limited number of times during surgery.

## 5 Conclusion

This study represents the first systematic investigation using protocols for a semi-quantitative evaluation of optic nerve microcirculation dynamics. It provided insights into the benefits of decompression on the ON and its microcirculation.

The immediate visual feedback provided by ICG-VA, in contrast to the delayed response of VEP, suggests a potential advantage in using ICG-VA for real-time assessments during surgery. However, the correlation with the clinical outcome across different tumor groups requires further refinement and investigation. The study proposes that combining these techniques could enhance understanding of the vascular supplies to both the optic nerve and the tumor, potentially leading to improved surgical outcomes.

Overall, the study lays the groundwork for future research, emphasizing the necessity of larger patient cohorts to validate the efficacy of ICG-VA as an intraoperative diagnostic tool in skull base surgeries aimed at vision preservation. This research could ultimately contribute to better surgical strategies and improved patient outcomes in the treatment of perichiasmatic pathologies.

## Data Availability

The original contributions presented in the study are included in the article/Supplementary material, further inquiries can be directed to the corresponding author.

## Supplementary material

SUPPLEMENTARY VIDEO 1 The video demonstrated case 5, involving a patient with perioptic meningioma. Initially, the ICG-VA before tumor removal indicated almost no ICG flow in the ON, explaining the absence of a peak ICG intensity in the ON. After tumor removal, the pial circulation of the ON significantly improved. At the end, a Flow 800 analysis sample was included following aneurysm clipping. ROI 1 represented the ICA, ROI 2 the ON, and ROI 3 the brain parenchyma slope.
